# Fabrication and Characterization of Dissolving Microneedles Containing *Oryza sativa* L. Extract Complex for Enhancement of Transfollicular Delivery

**DOI:** 10.3390/polym16162377

**Published:** 2024-08-22

**Authors:** Tanpong Chaiwarit, Baramee Chanabodeechalermrung, Pensak Jantrawut, Warintorn Ruksiriwanich, Mathukorn Sainakham

**Affiliations:** 1 Department of Pharmaceutical Sciences, Faculty of Pharmacy, Chiang Mai University, Chiang Mai 50200, Thailand; tanpong.ch@cmu.ac.th (T.C.); barameechana@gmail.com (B.C.); pensak.j@cmu.ac.th (P.J.); warintorn.ruksiri@cmu.ac.th (W.R.); 2 Center of Excellence in Materials Science and Technology, Chiang Mai University, Chiang Mai 50200, Thailand; 3 Center of Excellence in Agro Bio-Circular-Green Industry (Agro BCG), Agro-Industry, Chiang Mai University, Chiang Mai 50100, Thailand; 4 Cluster of Valorization and Bio-Green Transformation for Translational Research Innovation of Raw Materials and Products, Chiang Mai University, Chiang Mai 50200, Thailand

**Keywords:** dissolving microneedles, *Oryza sativa* L. extract complex, transfollicular penetration

## Abstract

Dissolving microneedles are extensively applied in drug delivery systems to enhance penetration into the skin. In this study, dissolving microneedles fabricated from polyvinylpyrrolidone K90 (PVP-K90) and hydroxypropylmethyl cellulose (HPMC) E50 in different ratios were characterized. The selected formulations incorporated *Oryza sativa* L. extract complex and its characteristics, transfollicular penetration, and safety were observed. The microneedles, fabricated from PVP K90: HPMC E50 in a ratio of 25:5 (P25H5) and 20:10 (P20H10), revealed excellent morphological structure, proper mechanical strength, and excellent skin insertion. P25H5 microneedles exhibited faster dissolution than P20H10 microneedles. Microneedles containing *Oryza sativa* L. extract complex showed excellent morphological structure via scanning electron microscopy but decreased mechanical strength. P25H5-O, which exhibited an effective ability to enter skin, was selected for further investigation. This microneedle formulation had a high percentage of drug-loading content, enhanced skin penetration via the transfollicular route, and was safe for keratinocytes. As a result, the dissolving microneedle containing *Oryza sativa* L. extract complex can be used to enhance transfollicular delivery through the skin with safety.

## 1. Introduction

Dermal drug delivery is a preferred method to administrate drugs for the treatment of skin-localized diseases and hair loss. This delivery system offers high local drug concentration, reduced systemic exposure, avoidance of hepatic first-pass metabolism, and improved patient compliance [1]. However, the penetration of most therapeutic drugs or active compounds into the skin is inhibited by the barrier function of the stratum corneum [2]. Microneedles are an effective option to resolve this problem because they can break the barrier of the stratum corneum to deliver therapeutics into the skin [3]. A microneedle is a small device consisting of numerous microscale needles in various shapes, arranged on a base support. Typically, the height of a microneedle ranges from 25 to 2000 µm [4]. Microneedles are used in drug delivery systems because they offer several advantages, such as avoiding first-pass metabolism and improving drug bioavailability, enhancing drug delivery to the skin, functioning as a painless device, increasing patient compliance, and controlling drug release [5,6,7]. Theoretically, microneedles create multiple micro-holes by piercing the skin, enabling the delivery of drugs or large substances through these micro-holes. Microneedles are categorized into four types: solid, hollow, coated, and dissolving microneedles [3]. Basically, dissolving microneedles are composed of water-soluble matrix materials such as hyaluronic acid, chondroitin sulfate, polyvinyl pyrrolidone (PVP), and hydroxypropyl methyl cellulose (HPMC), with therapeutic compounds dispersed or dissolved within the needles [3,8]. When applied to the skin, dissolving microneedles dissolve upon contact with biological fluid and release the loaded therapeutic compounds, which are then delivered directly to the therapeutic site [9]. PVP and HPMC are biodegradable, biocompatible, and non-toxic polymers that are suitable for skin insertion. Several dissolving microneedles have been fabricated from PVP and/or HPMC to deliver therapeutic compounds such as insulin, lidocaine, and alpha-arbutin [10,11,12]. Thus, the dissolving microneedle, fabricated from PVP and HPMC, might effectively deliver therapeutic or bioactive compounds for the treatment of hair loss or alopecia.

The development of an efficient delivery system is dependent on the transdermal penetration of topically applied compounds through human skin. Since the total amount of hair and its follicles represented only 0.1% of the total skin surface, they appear to be a significant target for drug delivery [13]. Delivery through hair follicles appears to depend on the distribution of hair follicle density throughout different parts of the body. Hair follicles have been shown to serve as a long-term reservoir for the kinetics of topically applied molecules and skin penetration pathways [14]. Numerous dermatological conditions, including acne, alopecia areata, and androgenetic hair loss, have been demonstrated to originate at the hair follicles [15]. In a recent study, it was demonstrated that the unsaturated fatty acids present in *Oryza sativa* L. have anti-hair loss effects by inhibiting the 5a-reductase enzyme in androgen-responsive organs [16]. A component of *Oryza sativa* L. extract complex was derived from byproduct extracts of four Thai rice types: Pieisu 1 CMU, Khao Dawk Mali 105, Bue Bang 3 CMU, and Bue Bang 4 CMU, which are herbal sources for hair growth-promoting substances [17,18,19,20].

In a previous study, the parameters in microneedle patch preparation that impact the appearance of the PVP/HPMC microneedle and the effects of different shapes on the microneedle’s characteristics were investigated. However, neither the characteristics of microneedles containing active compounds nor their skin penetration was investigated [17]. In the present study, we fabricated and characterized dissolving microneedles made from polyvinylpyrrolidone K90 (PVP-K90) and hydroxypropylmethyl cellulose E50 using 3D printing. The selected formulation of dissolving microneedle, which exhibited proper physicochemical properties, was incorporated into *Oryza sativa* L. extract complex to investigate its characteristics, transfollicular penetration, and safety. 

## 2. Materials and Methods

### 2.1. Materials

Polyvinylpyrrolidone K90 (PVP-K90) in granule form was purchased from MySkinRecipes (Bangkok, Thailand). Hydroxypropylmethyl cellulose E50 (HPMC E50) was purchased from ONIMAX (Bangkok, Thailand). Anhydrous ethanol was purchased from RCI Labscan (Bangkok, Thailand). 3-(4,5-Dimethylthiazol-2-yl)-2,5-diphenylte-trazolium bromide (MTT) was purchased from Sigma-Aldrich, Saint Louis, MO, USA. *Oryza sativa* L. extract complex solution (Hair Rise Complex^®^) was purchased from P2A innovation, Chiang Mai, Thailand. Deionized water (DI) served as a solvent to prepare polymeric solutions.

### 2.2. Fabrication of Microneedle Patch (Master Mold) Reverse PDMS Microneedle Mold

**Figure 1:**
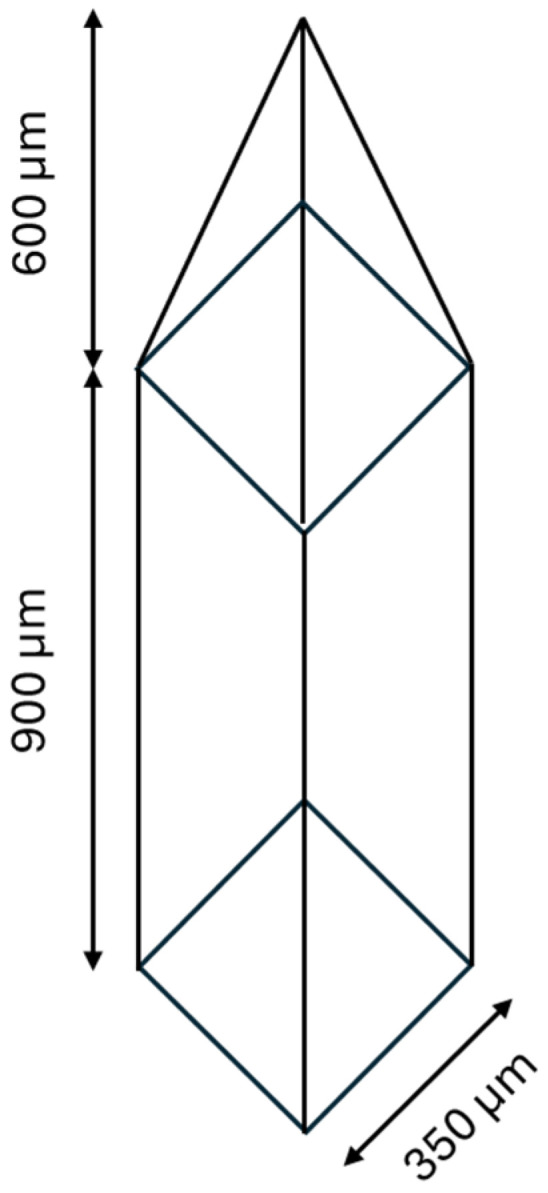
Design of the needle’s shape.

The microneedle was designed in the shape of a pyramid mounted on top of a long cube (Figure 1). The designed microneedle patch (master mold) was composed of an array of 15 × 15 needles with a 450 μm space between each needle. The methods to fabricate the microneedle patch and PDMS microneedle mold are described in a previous study [21]. The microneedle patch was designed using AUTODESK^®^ FUSION 360TM Software version 2.0.16985 (Autodesk Incorporation, San Rafael, CA, USA). The designed microneedle patch was 3D-printed using the LCD-based SLA 3D Printer (ANYCUBIC Photon, Anycubic Technology, Hong Kong, China). The microneedle patch was printed at a 30° angle pattern with curing times of 2.0 s and anti-aliasing. The acrylic-based resin (eResin PLA biophotopolymer resin, eSun, Shenzhen, China) was used to print the microneedle patch. After the printing process, the microneedle patch was washed with isopropyl alcohol to remove residual resin and cured with the ANYCUBIC Cure Machine 2.0 ultraviolet (UV) light-emitting diode (LED) lamp (Anycubic Technology, Hong Kong, China) for 20 min to solidify the microneedle patch. The microneedle patch was used to prepare a reverse polydimethylsiloxane (PDMS) microneedle mold. To fabricate a reverse PDMS mold, liquid-phase PDMS was mixed with curing agent at a ratio of 10:1 and poured into the microneedle patch. The bubbles inside the liquid-phase PDMS were removed using a vacuum pump (Trivac D16T, Leybold Dresden GmbH, Dresden, Germany). The PDMS was cured in a hot-air oven at 60 °C for 4 h. Finally, the reverse PDMS mold was gently removed from the microneedle patch.

### 2.3. Preparation of PVP K90/HPMC E50 Microneedles

PVP K90 and HPMC E50 were dissolved in an anhydrous ethanol/deionized water 70:30 solution to prepare the following formulations of PVP K90 and HPMC E50 in different ratios: 25:5, 20:10, 15:15, 10:20, and 5:25. The amount of each component in the formulations is shown in Table 1. The solutions were then stirred at 60 °C until they became homogenous. After that, the polymeric solutions were transferred to the centrifuge tubes, and the microneedle molds (reverse PDMS mold) were placed in the centrifuge tubes. The solutions were centrifuged at 8000 rpm at 25 °C for 1 h using a centrifuge machine (MPW-352R, Warsaw, Poland) to ensure the solution filled the mold cavity. Then, the microneedle molds were taken out of the centrifuge tubes and heated at 45 °C until the microneedles dried. Finally, the dried microneedles were carefully removed from the molds.

### 2.4. Morphological Structure Investigation

The microneedle structures were examined for the morphologies and dimensions using an RS PRO USB digital microscope (RS PRO, Bangkok, Thailand) and a scanning electron microscope (JEOL LCM7000 NeoScopeTM Benchtop, Tokyo, Japan) at 15 kV in low vacuum mode. The prepared microneedles were cut and attached to an aluminum tab with double-sided adhesive carbon tape. All the microneedles were gold-sputtered coated for 1 min prior to the SEM imaging at 50× magnification. The heights and widths of the microneedles were analyzed using Image J software version 1.80, and the fabrication accuracy percentage (% accuracy) of the microneedles was calculated using the following Equations (1) and (2).

(1) $$\mathrm{Height}\;\mathrm{accuracy}\;(\%)=\frac{\mathrm{Actual}\;\mathrm{height}\;(µm)}{\mathrm{Designed}\;\mathrm{height}\;(µm)}\times 100$$

(2) $$\mathrm{Width}\;\mathrm{accuracy}\;(\%)=\frac{\mathrm{Actual}\;\mathrm{width}\;(µm)}{\mathrm{Designed}\;\mathrm{width}\;(µm)}\times 100$$

### 2.5. Fourier Transform Infrared Spectroscopy (FTIR)

The microneedles were investigated for interaction between each component using a Fourier transform infrared (FTIR) spectrometer (FT/IR-4700, Jasco, Tokyo, Japan). The microneedles and materials were scanned in transmittance mode from 400 to 4000 cm^−1^ at a resolution of 4 cm^−1^.

### 2.6. Mechanical Strength

The method to measure the mechanical strength of the microneedles was adapted from a previous study [22]. The mechanical strength of the microneedles was investigated using a Texture Analyzer TA.XTplus (Stable Micro Systems, Surrey, UK) equipped with a load cell (5 kg) with a P/25 flatted stainless steel probe in compression mode. The compression test was performed at a rate of 0.1 mm/s for a distance of 2.0 mm. The forces of compression at 500 µm were recorded and compared for each microneedle formulation. Before and after the test, the height of the microneedles was photographed using an RS PRO USB digital microscope (RS PRO, Bangkok, Thailand) and measured using ImageJ software version 1.80. The percentage change in height was calculated using Equation (3).

(3) $$\%\;\mathrm{Height}\;\mathrm{change}=\frac{H_{1}-H_{2}}{H_{1}}\times 100$$

where

*H*_1_ is the initial height of the microneedle (µm);
*H*_2_ is the height of the microneedle after the test (µm).

### 2.7. Differential Scanning Calorimetry (DSC) Studies

The crystallinity of the polymers and microneedles was investigated using a differential calorimeter (DSC25, TA instrument, New Castle, DE, USA). The test was performed under a nitrogen gas environment. The temperature increased from 20 to 250 °C at a scanning rate of 10 °C/min [23].

### 2.8. Ex Vivo Skin Insertion Test

The method for investigating skin insertion was adapted from previous studies [11,24]. Neutral death piglets (stillborn) were collected from a local industrial pig farm in Lamphun Province, Thailand. Before the test, neonatal porcine skin without a subcutaneous layer was gently removed and washed with phosphate-buffered saline (PBS), pH 7.4. The hair on the neonatal porcine skin was gently shaved before the test. Microneedles were pressed into the skin using a thumb for 1 min. Then the microneedles were removed, and methylene blue solution (0.1%) was applied to the skin for 10 min immediately. After that, the methylene blue solution was washed off with PBS, pH 7.4. Finally, the dyed pores (blue dots) were counted, and the blue-dot percentage was calculated using Equation (4).

(4) $$\%\;\mathrm{Blue}\;\mathrm{dots}=\frac{\mathrm{The}\;\mathrm{number}\;\mathrm{of}\;\mathrm{blue}\;\mathrm{dots}}{\mathrm{Total}\;\mathrm{number}\;\mathrm{of}\;\mathrm{needles}\;}\times 100$$

### 2.9. Dissolution of Microneedles

The method to investigate the dissolution of microneedles was adapted from a previous study [25]. Neonatal porcine skins were saturated with PBS, pH 7.4. The hairs of the neonatal porcine skins were gently shaved before the test. Microneedles were applied and kept on the neonatal porcine skins without a subcutaneous layer for 0, 5, 15, 30, and 45 min. The microneedles were removed at each predetermined interval, and the change in microneedle structure before and after the dissolution study was examined by an RS PRO USB digital microscope (RS PRO, Bangkok, Thailand).

### 2.10. Preparation and Characterization of Microneedles Containing Oryza sativa L. Extract Complex

To load *Oryza sativa* L. extract complex into the microneedles, the proper microneedle formulations were selected based on physical appearance, mechanical strength, and ex vivo skin insertion. The *Oryza sativa* L. extract complex was added to the polymeric solution at a concentration of 40% *w*/*w* based on dried polymer. The microneedles containing *Oryza sativa* L. extract complex were prepared by the method described in Section 2.2. The microneedles containing *Oryza sativa* L. extract complex were characterized using the methods previously described.

### 2.11. Drug-Loading Content

**Figure 2:**
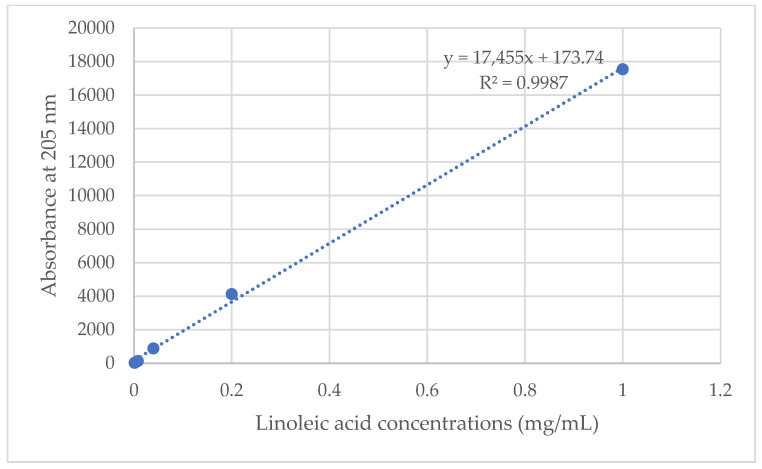
The standard curve graph of linoleic acid for drug-loading content determination.

The microneedles containing *Oryza sativa* L. extract complex were dissolved in a test tube with 100% methanol (1 mL) using an ultrasonic cleaner (Elmasonic S100 H, JP-50 kHz, 550 W; Elma, Germany) for 30 min and filtrated through a 0.2 μm cellulose acetate membrane filter. After that, the average amount of linoleic acid in *Oryza sativa* L. extract complex was examined using HPLC under the same conditions, with the wavelength of maximum absorption at 205 nm, as previously studied [26]. The contents of linoleic acid were calculated from the standard curve of linoleic acid (1.6–1000 μg/mL), with a high linear regression (r^2^ = 0.9987), as shown in Figure 2 and according to Equation (5).

(5) $$\%\;\mathrm{Drug}\;\mathrm{loading}\;\mathrm{content}=\frac{\mathrm{The}\;\mathrm{amount}\;\mathrm{of}\;\mathrm{linoleic}\;\mathrm{acid}\;\mathrm{in}\;\mathrm{microneedle}\;\mathrm{patch}}{\mathrm{Theoretical}\;\mathrm{amount}\;\mathrm{of}\;\mathrm{linoleic}\;\mathrm{acid}\;\mathrm{in}\;\mathrm{dry}\;\mathrm{polymer}}\times 100$$

### 2.12. Transfollicular Penetration Studies of Skin by Franz Diffusion Cells

Male newborn landrace pigs that died of natural causes a few days after birth were obtained from a Betagro Northern Agro-Industry Co., Ltd., farm in Lamphun, Thailand. The preparation of the skin sample was performed as previously mentioned [16]. The back pig skin was meticulously separated following the shaving process. The subsequent step involved the use of a scalpel to precisely remove the excess subcutaneous fat using the pinching technique. A drop of power glue (99.0%, *w*/*w* cyanoacrylate) was applied to the skin and subsequently covered with a glass slide under mild pressure. After 5 min of cyanoacrylated polymerization, the glass slide was rapidly removed to reveal all hair follicles. The number of hair follicles on the porcine skin was counted. A pair of skin samples with an equal amount of hair follicles was used as opened and blocked hair follicle skin samples. The follicular closing technique was employed to close all hair follicles in the obstructed hair follicle skin. The follicular orifices of the blocked hair follicle skin sample were carefully blocked with a small amount of nail vanish using a blunt 30 gauge needle (Nipro, Bangkok, Thailand). The area was then dried for 5 min to ensure that the follicular shunt was effectively closed. Transfollicular delivery by vertical Franz diffusion cells in a microneedle and a solution sample of the extract containing linoleic acid as the marker was performed by the follicular closing technique [27]. The viable epidermis side of the blocked and opened hair follicle skins was mounted upwards to the donor compartment, while the dermal side was in contact with the receiver. The donor and receiver chambers had a contact area of 1.77 cm^2^. The temperature was maintained at 37 ± 2 °C, and the receptor compartment (phosphate-buffered saline (PBS), pH 7.4) was continuously stirred at 100 rpm with a magnetic bar throughout the experiment. The volume of the compartment was 13 mL. A paraffin film was used to cover 200 μL of PBS added with a microneedle containing the extract or the extract solution in the donor compartment. Cells were stopped at 15, 30, and 45 min. The treated porcine skin samples were removed and washed twice with distilled water. The skin was stripped using 3 M Scotch^®^ Magic^TM^ tape (3M, Bangkok, Thailand) (1 cm × 1 cm). After 10 s of charging with a 200 g weight, each tape was rapidly removed to eliminate excess samples. The stripped skin was cut into small pieces, extracted with 2 mL of methanol, sonicated for 10 min, and filtered. The linoleic acid content in the skin was analyzed using HPLC, as previously described [26]. Three microneedles were tested at each predetermined time separately in blocked and opened hair follicle conditions. The cumulative amounts, fluxes, and transfollicular penetration per hair follicle (HF) in the skin were determined using the following Equations (6)–(8).

(6) $$\mathrm{Cumulative}\;\mathrm{amount}\;(\mathrm{ng}/{\mathrm{cm}}^{2})=\frac{\mathrm{LAopen}-\mathrm{LAblock}}{1.77}$$

(7) $$\mathrm{Fluxes}\;(\mathrm{ng}/{\mathrm{cm}}^{2}/\min)=\frac{\mathrm{LAopen}-\mathrm{LAblock}}{1.77\times \min}$$

(8) $$\mathrm{Transfollicular}\;\mathrm{penetration}\;\mathrm{per}\;\mathrm{one}\;\mathrm{hair}\;\mathrm{follicle}\;(\mathrm{ng}/\mathrm{HF})=\frac{\mathrm{LAopen}-\mathrm{LAblock}}{\mathrm{Number}\;\mathrm{of}\;\mathrm{HF}}$$

where LAopen is linoleic acid amounts in the opened hair follicle skin, LAblock is linoleic acid amounts in the blocked hair follicle skin, and HF is the hair follicle.

### 2.13. Cell Viability Assay

An immortalized human epidermal keratinocyte cell line (HaCaT) was obtained from Cell Lines Service GmbH (Eppelheim, Baden-Württemberg, Germany). To culture the HaCaT cells, the cells were grown in Dulbecco’s Modified Eagle Medium (DMEM) (Thermo Fisher Scientific, Waltham, MA, USA) with 10% fetal bovine serum (Merck KGaA, Darmstadt, Germany) as a supplement. The antibiotics (100 U/mL penicillin and 100 μg/mL streptomycin) (Thermo Fisher Scientific, Waltham, MA, USA) were added to prevent bacterial contamination. The culture was incubated under humidified conditions at 37 °C with 5% CO_2_.

The toxicity of microneedles containing *Oryza sativa* L. extract complex to HaCaT cells was investigated by MTT assay. The method was adapted from a previous study [28]. HaCaT cells were seeded in DMEM in 96-well plates at a density of 8000 cells per well and incubated for 24 h. The microneedles were sterilized by UV radiation for 30 min. Ten milligrams of the microneedles, both with and without *Oryza sativa* L. extract complex and *Oryza sativa* L. extract complex solution, were added to 10 mL of DMEM and kept in the refrigerator for 24 h, then filtered through a 0.2 µm syringe filter. The samples were diluted with DMEM to obtain concentrations of 6.25, 12.5, 25, 50, 100, 250, 500, and 1000 µg/mL. HaCaT cells were treated with 200 µL of each sample at these concentrations for 24 h. Two hundred microliters of MTT reagent (0.4 mg/mL) were added to each well and incubated for 2 h. The supernatants of cultures were removed, and 100 µL of dimethyl sulfoxide (DMSO) was added to solubilize the formazan complex. Finally, the absorbance of the samples was measured at 570 nm using a microplate reader (BioTek Instruments, Winooski, VT, USA). The percentage of cell viability was calculated using Equation (9).

(9) $$\mathrm{Cell}\;\mathrm{viability}\;(\%)=\frac{\mathrm{Absorbance}\;\mathrm{of}\;\mathrm{treatment}\;\mathrm{at}\;570\;\mathrm{nm}}{\mathrm{Absorbance}\;\mathrm{of}\;\mathrm{control}\;\mathrm{at}\;570\;\mathrm{nm}}\times 100$$

### 2.14. Statistical Analysis

The significant difference between the results was investigated at a significance level of 0.05 by SPSS software (version 17; IBM corporation, New York, NY, USA). Statistically significant differences between sample groups of the results were analyzed by one-way analysis of variance (ANOVA), followed by LDS’s post hoc test.

## 3. Results and Discussion

### 3.1. Morphological Structure of Microneedles

**Figure 3:**
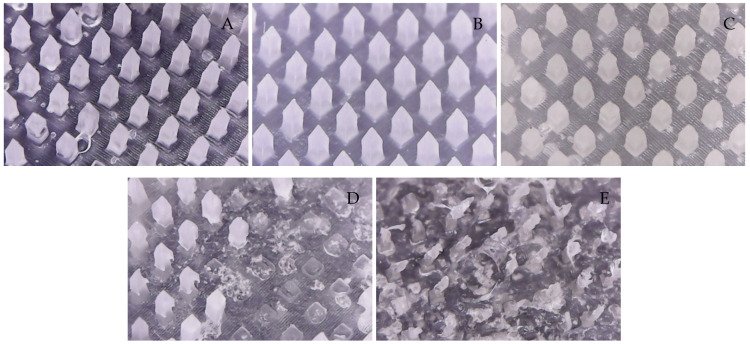
Images from the digital microscope showing the needle physical morphology of P25H5 (**A**), P20H10 (**B**), P15H15 (**C**), P10H20 (**D**), and P5H25 (**E**).

**Figure 4:**
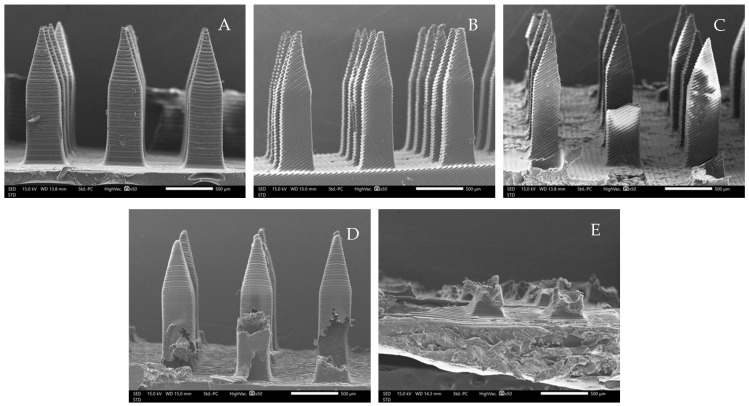
SEM micrographs showing the needle physical morphology of P25H5 (**A**), P20H10 (**B**), P15H15 (**C**), P10H20 (**D**), and P5H25 (**E**).

The microneedle structures were examined using the digital microscope and SEM at 50× magnification to select the formulation with a desirable physical appearance. This study found that the amount of PVP K90 played a pivotal role in the microneedle structure. To clarify, the less PVP K90, the less accordance with the theoretical geometries of the master molds. According to images from the digital microscope and SEM micrographs in Figure 3 and Figure 4, the microneedle structures of P25H5 and P20H10, which had high amounts of PVP K90, revealed fully formed needles with sharp tips in the array. In contrast, microneedles with lower PVP K90, such as P15H15, P10H10, and P5H25, exhibited tip bending. Consequently, P15H15, P10H10, and P5H25 had a lower percentage of the accuracy of height (Table 2). Furthermore, the P10H10 and P5H25 exhibited unformed needles with several holes at the microneedle bases. These microneedles were not desirable, as shown in Figure 3C–E and Figure 4C–E, because of an unsuitable physical structure such as broken tips, incomplete needles, and irregular shapes, which led to poor mechanical properties and affected skin penetration [29]. Consistent with these observations, the % accuracy of height and width for P25H5 and P20H10 was close to 100%, with low standard deviations (98.63 ± 1.27% and 98.33 ± 1.20% for height, and 101.41 ± 1.65% and 86.93 ± 1.74% for width), indicating better consistency and closer adherence to the desired dimensions compared to the formulations with lower PVP K90. Previous studies have also successfully fabricated PVP-blended microneedles; for example, microneedles made from PVA and PVP closely matched theoretical dimensions [24].

### 3.2. Fourier Transform Infrared Spectroscopy (FTIR)

**Figure 5:**
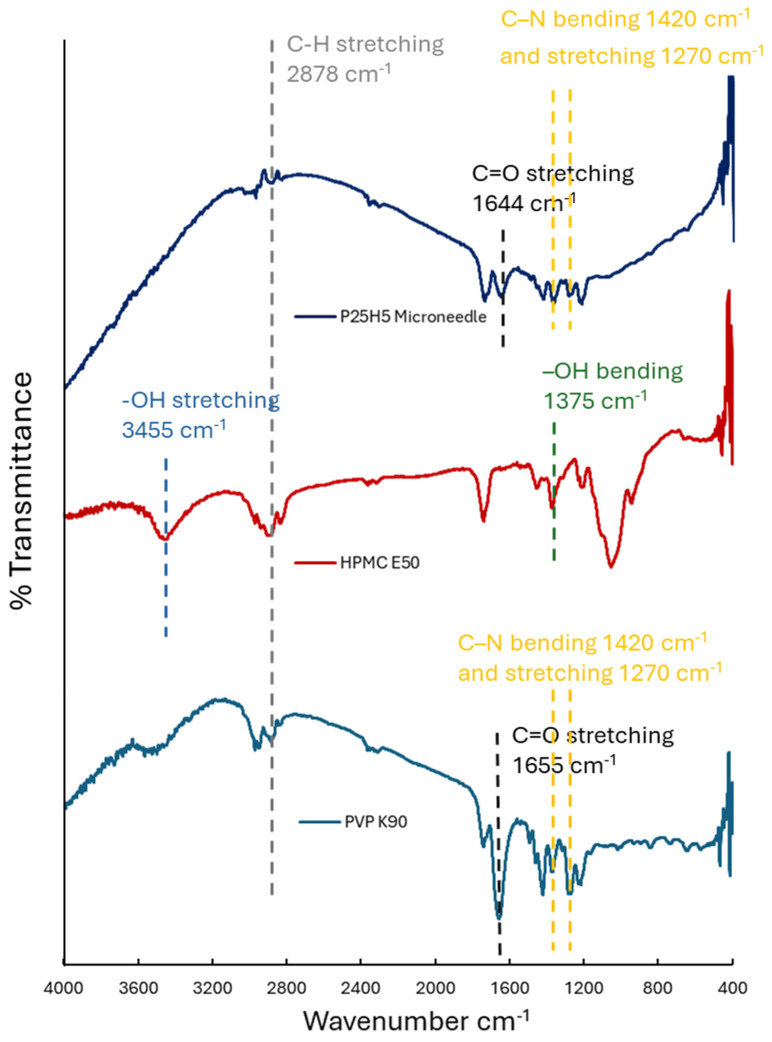
FTIR spectra of PVP K90, HPMC E50, and P25H5 microneedle.

FTIR spectra were utilized to investigate chemical interactions between PVP K90 and HPMC E50 in microneedles. The FTIR spectra of PVP K90, HPMC E50, and P25H5 microneedles are shown in Figure 5. The FTIR spectrum of PVP K90 displayed dominant functional groups related to its chemical structure. The peak at 2878 cm^−1^ represents C–H stretching. The high-intensity peak at 1655 cm^−1^ corresponds to C=O stretching of carbonyl groups in the pyrrolidone ring. Two peaks at 1420 and 1270 cm^−1^ are associated with C–N bending and stretching, respectively [30,31,32]. The FTIR spectrum of HPMC E50 showed C–H stretching at 2878 cm^−1^ and important peaks indicating –OH stretching and bending at 3455 and 1375 cm^−1^, respectively [30,33,34]. The pattern of FTIR spectrum of the P25H5 microneedle is consistent with VPV K90 and HPMC E50. Interestingly, the peak of –OH stretching of HPMC E50 at 3455 cm^−1^ was absent, and the peak of C=O of PVP K90 slightly shifted from 1655 to 1644 cm^−1^ and decreased in intensity. These indicate a possible interaction between PVP K90 and HPMC E50 in the microneedle because the C=O groups in the pyrrolidone ring of PVP could form hydrogen bonds with –OH groups of HPMC [35].

### 3.3. Mechanical Test

The mechanical strength of microneedles is related to their ability to pierce through the epidermal layer without breaking. A texture analyzer is widely used to preliminarily determine the hardness or fragility of microneedles during compression [29]. Both microneedle formulations exhibited proper compression force, as shown in Table 3. At a displacement of 500 µm, the compression forces of the P25H5 and P25H10 microneedles were 41.94 ± 3.06 N/array (0.19 ± 0.01 N/needle) and 31.60 ± 1.60 N/array (0.14 ± 0.01 N/needle), respectively. The force required for reliable skin penetration is around 0.058 N/needle [36,37]. Therefore, the mechanical strength of the P25H5 and P25H10 microneedles was sufficient for effective skin penetration. The force value of the P25H5 microneedle (N/needle) was significantly higher than that of the P20H10 microneedle, indicating that the addition of the PVP K90 ratio in the formulation increased the mechanical strength. Basically, PVP K90 is hard and tough. In previous studies, the addition of PVP K90 to microneedles enhanced mechanical strength and reduced fragility [3,38].

The percentage of height change reflects the mechanical strength of the microneedles. Previous studies reported that microneedles with great mechanical strength should have a percentage height change of less than 10% [39,40]. This indicates that microneedles retain their shape during compression without bending or breaking. The P25H5 and P25H10 microneedles exhibited 3.40 ± 2.04 and 7.89 ± 1.74% height changes, respectively. Although both formulations showed good percentage height, the percentage height change of P25H5 was significantly lower than that of P25H10. According to the compression force, P25H5 showed a higher force value than P20H10. In addition, the addition of PVP K90 to the formulation increased the hardness of the microneedles [3,38,41].

### 3.4. Differential Scanning Calorimetry (DSC) Studies

**Figure 6:**
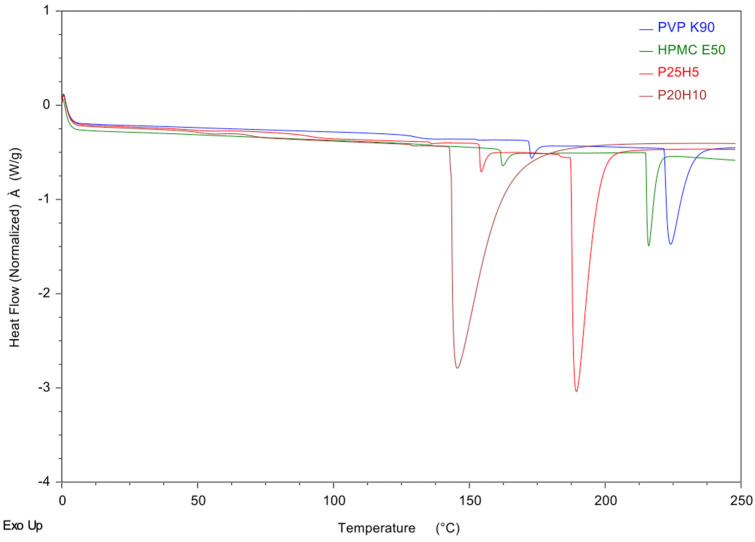
DSC thermograms of the PVP K90, HPMC E50, P25H5, and P20H10 microneedles.

The DSC thermograms of the PVP K90, HPMC E50, P25H5, and P20H10 microneedles are shown in Figure 6. PVP K90 and HPMC E50 exhibited strong endothermic peaks at 226 and 216 °C, respectively, while the endothermic peaks of the P25H5 and P20H10 microneedles were lower than PVP K90 and HPMC E50 (189 and 145 °C for P25H5 and P20H10, respectively). As the concentration of PVP K90 increased, the endothermic peak of the microneedle increased. This indicates that PVP K90 improved the thermal stability of the microneedles due to the interaction between PVP K90 and HPMC E50 [42]. Compared to pure PVP K90 and HPMC E50, the endothermic peaks of the P25H5 and P20H10 microneedles were wider. This indicates that the crystalline structure of PVP K90 and HPMC E50 was destroyed and turned amorphous [42,43]. In addition, the endothermic temperature of P25H5 was higher than the P20H10 microneedle. This indicates that increasing the PVP ratio increased the crystalline structure of the microneedles [42]. It is possible that P25H5 displayed a higher mechanical strength than the P20H10 microneedle.

### 3.5. Ex Vivo Skin Insertion Test

**Figure 7:**
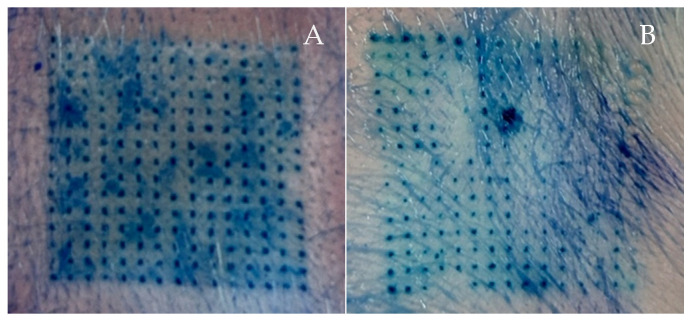
Skin insertion capability of P25H5 (**A**) and P20H10 (**B**).

Skin penetration is an important ability of microneedles. A skin insertion test was conducted to ensure that the microneedles could pierce through the epidermal layer [29]. To investigate the types and amounts of polymers that influence the skin insertion capability of microneedles, the percentage of blue dots was calculated based on the blue dots that appeared on neonatal porcine skin. The blue dots represent the insertion of microneedles into the skin [3]. To clarify, a higher number of blue dots indicates more successful skin penetration. In this study, due to their good morphological structure, the P25H5 and P20H10 microneedles were chosen for an ex vivo skin penetration test. The skin insertion capability of the P25H5 and P20H10 microneedles is shown in Figure 7 and Table 3. There were significantly more blue dots in P25H5 (98.52 ± 1.43%) than in P20H10 (95.26 ± 2.45%). Based on the mechanical strength, the P25H5 microneedle exhibited a higher compression force than P20H1; thus, it could provide a higher percentage of blue dots. This indicates that a higher PVP K90 content enhanced the strength and hardness of the microneedles, increasing their ability to penetrate the skin and resulting in a higher percentage of blue dots. PVP K90 was a biocompatible polymer with excellent toughness and hardness. Hence, it was used to improve the mechanical strength and friability of the microneedles [9,12]. These properties of PVP K90 contribute to better skin insertion of microneedles.

### 3.6. Dissolution of Microneedles

The P25H5 and P20H10 formulations were selected for the dissolution study based on their optimal morphologies. The dissolution study showed the change in microneedle structure over time at 0, 5, 15, 30, and 60 min, as shown in Table 4. Interestingly, P25H5 microneedles exhibited faster dissolution; their tips began to visually bend after 5 min and were completely dissolved by 45 min. On the other hand, P20H10 microneedles started dissolving after 15 min, and even after 45 min, few needles remained, indicating that the microneedles did not dissolve thoroughly. This result could be explained by the hygroscopic properties of the polymers. Generally, PVP and HPMC are hydrophilic and water-soluble polymers [44,45]. The microneedles consisting of higher amounts of PVP K90 exhibited faster dissolution because PVP K90 had a better ability to adsorb moisture from neonatal porcine skin to dissolve microneedles than the microneedles with a lower PVP K90 ratio. In addition, a previous study reported that the increase in the PVP ratio in dissolving microneedles reduced the time required for dissolution and created a hole in the microneedles with water [38]. Hydrogen bonding might also impact the dissolution of microneedles. The hydrogen bonding between the carbonyl groups of PVP and the hydroxyl groups of HPMC is stronger when the amount of HPMC increases [46]. Probably, the higher-ratio HPMC microneedle (P20H10) exhibited slower dissolution than the lower-ratio HPMC microneedle (P25H5). The strength of hydrogen bonds also affected the release of incorporated active compounds (*Oryza sativa* L. extract) because the release of incorporated compounds from dissolving microneedles depends on the dissolution of the microneedles. In other words, theP25H5 microneedle probably releases *Oryza sativa* L. extract faster than the P20H10 microneedle. For these reasons, the P25H5 microneedle is more suitable for delivering active compounds with immediate release.

### 3.7. Characterization of Microneedles Containing Oryza sativa L. Extract Complex

**Figure 8:**
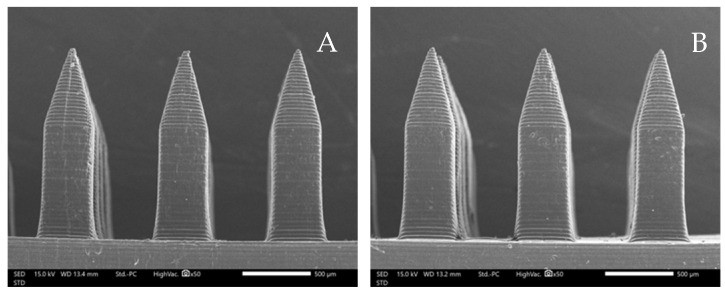
SEM micrographs showing the physical morphology of the P25H5-O (**A**) and P20H10-O (**B**) microneedles.

Due to the morphology and characteristics of the microneedles, P25H5 and P20H10 formulations were selected to incorporate *Oryza sativa* L. extract complex. The SEM micrographs of P25H5 and P20H10 microneedles containing *Oryza sativa* L. extract complex (P25H5-O and P20H10-O microneedles) are shown in Figure 8. Both microneedles possessed excellent morphological structures. The morphology and characteristics of the P25H5-O and P20H10-O microneedles are shown in Table 5. The P25H5-O and P20H10-O microneedles exhibited high accuracy of height (94.48 ± 0.43 and 94.35 ± 1.04, respectively). However, the needle height slightly decreased compared to formulations without *Oryza sativa* L. extract complex. The P25H5-O and P20H10-O microneedles also showed excellent accuracy of width. However, the accuracy of the width of P25H5-O (101.47 ± 1.44%) was more accurate than that of P20H10-O (91.43 ± 3.68%).

**Figure 9:**
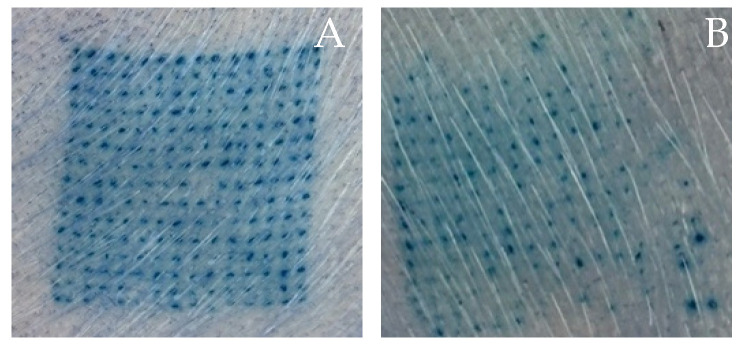
Skin insertion capability of the P25H5-O (**A**) and P20H10-O (**B**) microneedles.

The mechanical test revealed that the compression force of P25H5 and P20H10 significantly decreased from 41.94 ± 3.06 N/array (0.19 ± 0.01 N/needle) and 31.60 ± 1.60 N/array (0.14 ± 0.01 N/needle) to 26.52 ± 1.29 N/array (0.12 ± 0.01 N/needle) and 17.75 ± 2.03 N/array (0.08 ± 0.01 N/needle), respectively, when the *Oryza sativa* L. extract complex was added. This study indicated that the *Oryza sativa* L. extract complex probably acted as a plasticizer to increase flexibility and decrease mechanical strength. The *Oryza sativa* L. extract complex solution consisted of propylene glycol and glycerol as co-solvents to dissolve the active compounds, which are effective plasticizers for several polymers, including PVP and HPMC [47]. In addition, compared with the microneedles without *Oryza sativa* L. extract complex, the percentage height change of P25H5 and P20H10 increased significantly from 3.40 ± 2.04 and 7.89 ± 1.74% to 8.22 ± 1.82 and 16.30 ± 3.66%, respectively, due to tip bending after compression without breaking, especially for P20H10-O. Importantly, the P25H5-O microneedles exhibited significantly better skin insertion than the P20H10-O microneedles (93.04 ± 7.03 and 53.63 ± 13.31%, respectively) due to the higher compression force of P25H5-O (Table 5 and Figure 9). In contrast, P20H10-O, possessing lower mechanical strength and more tip bending, showed a lower percentage of blue dots. Based on these results, P25H5-O was selected for further investigation.

### 3.8. Drug-Loading Content

**Figure 10:**
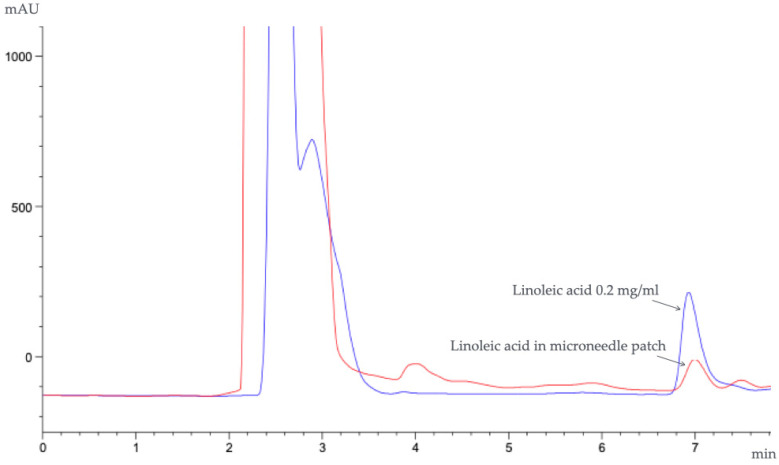
HPLC chromatogram of linoleic acid standard (blue line) and linoleic acid in the microneedles containing 40% *w*/*w Oryza sativa* L. extract complex (red line).

In a previous study, the major fatty acid compositions of several rice varieties were oleic acid, palmitic acid, and linoleic acid. It was also reported that the activities of 5α-reductases were inhibited by the bioactive constituent linoleic acid that can be found in *Oryza sativa* L. extract complex, resulting in inhibiting the conversion of testosterone to DHT and ameliorating progressive miniaturization [19]. Linoleic acid is a potent component that regulates the cell cycle by increasing cyclin D1 and CDK2 expression, stimulating the Wnt/β-catenin pathway, and promoting hair cell proliferation. A crucial characteristic of dihydrotestosterone-induced hair loss is reduced cell proliferation through Wnt/β-catenin signaling suppression. Linoleic acid stimulates Wnt/β-catenin signaling and suppresses DKK1 expression via dihydrotestosterone [48]. Therefore, linoleic acid was selected as an active marker in *Oryza sativa* L. extract complex and determined the content of microneedles in this study. The 40% *w*/*w Oryza sativa* L. extract complex concentration was selected for the studied formulations, and the added amount of the extract complex was calculated from the weight of the polymer in the formulation. The HPLC chromatogram of the linoleic acid standard at 0.2 mg/mL and the linoleic acid in the microneedles containing 40% *w*/*w* *Oryza sativa* L. extract complex are shown in Figure 10. It was found that the percentage of drug-loading content in the microneedles containing 40% *w*/*w Oryza sativa* L. extract complex was 92.95 ± 9.04%.

### 3.9. Transfollicular Penetration Test by Franz Diffusion Cells

**Figure 11:**
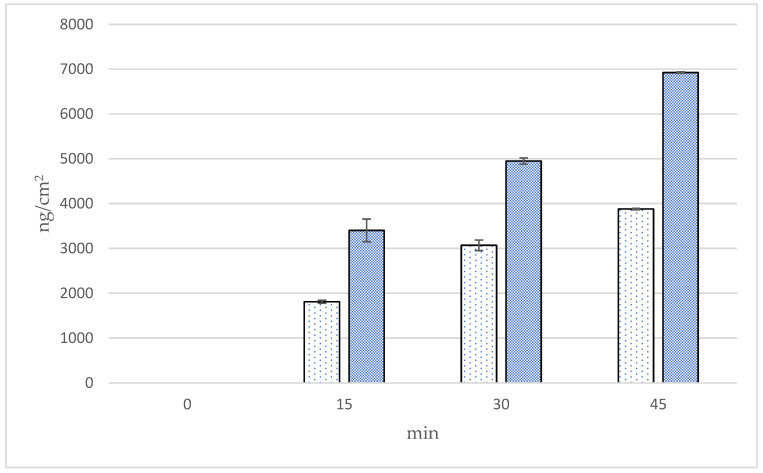
Cumulative amounts of linoleic acid in skin (ng/cm^2^) by the follicular closing technique using Franz diffusion cells at 0, 15, 30, and 45 min. 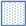 Solution formulation, 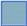 microneedle formulation.

**Figure 12:**
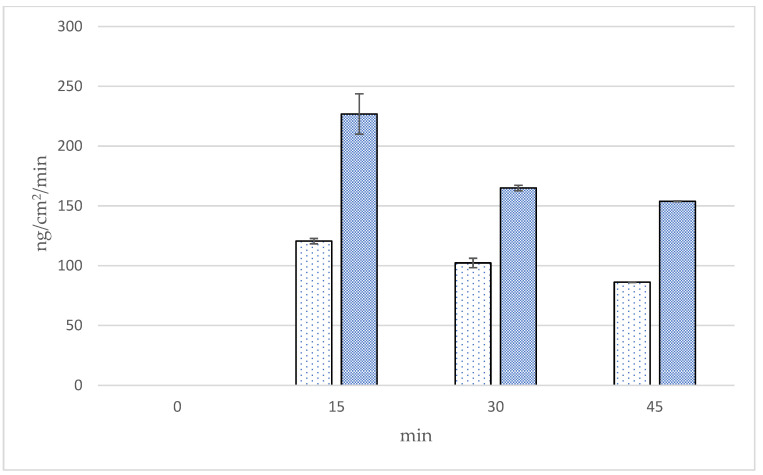
Fluxes of linoleic acid in skin (ng/cm^2^/min) by the follicular closing technique using Franz diffusion cells at 0, 15, 30, and 45 min. 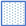 Solution formulation, 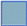 microneedle formulation.

**Figure 13:**
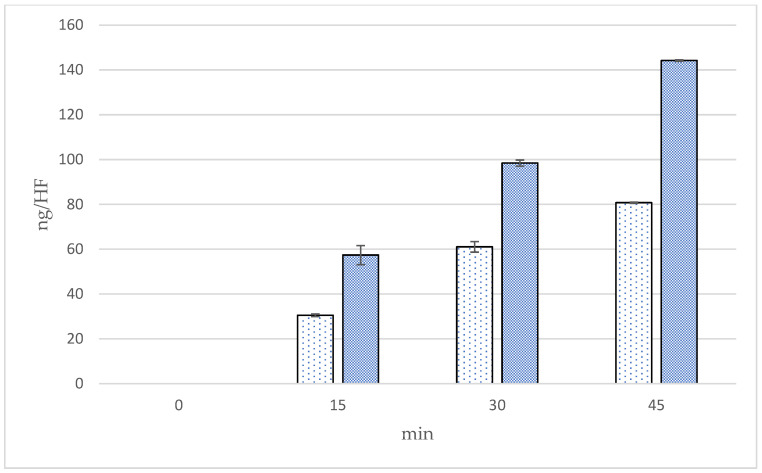
Follicular penetration per hair follicle of linoleic acid in skin (ng/HF) by the follicular closing technique using Franz diffusion cells at 0, 15, 30, and 45 min. 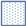 Solution formulation, 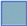 microneedle formulation.

The cumulative amounts, fluxes, and follicular penetration per hair follicle of linolenic acid using Franz diffusion cells in the skin at 0, 15, 30, and 45 min from two formulations of *Oryza sativa* L. extract complex are presented in Figure 11, Figure 12 and Figure 13 and Table 6. The cumulative amounts of linolenic acid in the skin from all formulations increased with time. After 45 min, microneedle formulation (9624.72 ± 33.65 ng/cm^2^) exhibited higher cumulative amounts of linoleic acid in the skin than solution formulation (3878.80 ± 33.25 ng/cm^2^), about 1.8 times. In microneedle formulation, the cumulative amounts of linolenic acid at 45 min were significantly higher than at 15 and 30 min (*p* < 0.05). The results also illustrate that the fluxes of linoleic acid in the skin from the microneedle formulation were higher than those from the solution formulation at 15, 30, and 45 min. Fluxes of linoleic acid in the skin from the microneedle formulation at 45 min were significantly higher than at 15 min (*p* < 0.05). Follicular penetration of linoleic acid per hair follicle had similar results as cumulative amounts of linoleic acid in the skin. Follicular penetration per hair follicle from the microneedle and solution formulation at 45 min was 144.20 ± 0.70 and 80.77 ± 0.53 ng, respectively, which was significantly different at *p* < 0.05. It is well established that there is no noticeable difference in the in vitro skin permeability between porcine and human skin. Porcine skin has been shown to have a similar structure to human skin, resulting in a suitable model for studying the penetration of substances through the skin [16]. A drug-loaded microneedle, a small device used in transdermal drug delivery systems, can temporarily penetrate the stratum corneum, improving skin permeability and subsequently enhancing penetration efficiency. Interestingly, microneedles are well known to be a painless device because they penetrate the epidermis layer to the upper dermis layer without facing the skin barrier [21]. For microneedle formulation, linoleic acid appeared to penetrate the skin more than solution formulation, resulting in higher transfollicular penetration per hair follicle and fluxes through hair follicles in this experiment. This indicates that the microneedle formulation may facilitate the transfollicular delivery of the active constituents. This study has suggested that the microneedle containing *Oryza sativa* L. extract complex appears to be a suitable system to be used in a transfollicular delivery system. 

### 3.10. Cell Viability Test

**Figure 14:**
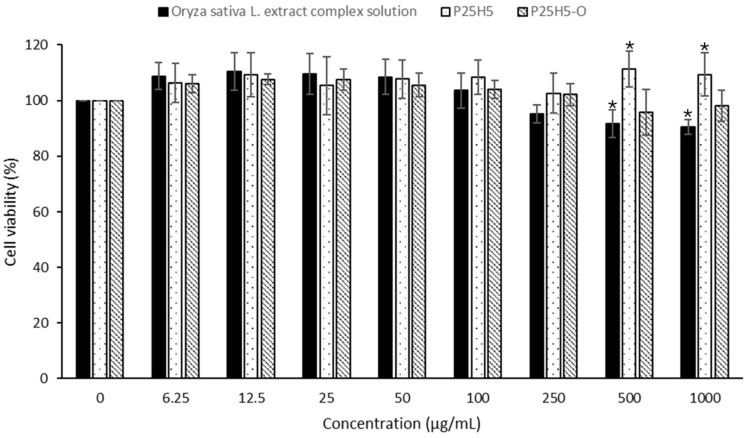
Cell (HaCaT) viability of *Oryza sativa* L. extract complex solution with P25H5 and P25H5-O microneedles. * Indicates significant different in the same concentration (*p* < 0.05).

To investigate the skin toxicity of the microneedles, the keratinocyte cell (HaCaT) was treated. The P25H5 and P25H5-O microneedles exhibited cell viability of more than 90%, with insignificant differences between each other in all concentrations (Figure 14). Although the *Oryza sativa* L. extract complex solution showed lower cell viability than the P25H5 microneedle, the difference was not significant compared to the P25H5-O microneedles. Generally, PVP K90 and HPMC E50 are non-toxic polymers. Linoleic acid, an active marker compound of *Oryza sativa* L. extract complex solution, is an essential component of the stratum corneum, more beneficial to skin health, and used in dermal and transdermal drug delivery [49]. Thus, for the *Oryza sativa* L. extract complex solution, the P25H5 and P25H5-O microneedles showed high % cell viability. This experiment indicated that P25H5-O microneedles are initially safe for application on the skin.

## 4. Conclusions

The microneedle structures with a high amount of PVP K90, P25H5, and P20H10 revealed fully formed needles with suitable morphological structures. The pattern of the FTIR spectrum of the microneedle exhibited a possible interaction between the C=O groups in the pyrrolidone ring of PVP K90 and hydrogen bonds with –OH groups of HPMC E50.

The mechanical strength of the P25H5 and P25H10 microneedles made it possible to effectively penetrate skin. Furthermore, the mechanical test showed that PPV K90 played an important role in increasing the mechanical strength as well as the hardness of the microneedles. Based on the DSC characterization, PVP K90 and HPMC E50 interactions increased microneedle thermal stability. P25H5 had a greater endothermic temperature than the P20H10 microneedle, implying that it possessed superior mechanical strength. In the ex vivo skin penetration test, the higher composition of PVP K90 enhanced the strength and hardness of the microneedles to increase the possibility of needles penetrating the skin, leading to more blue dots in the P25H5 microneedle than in the P20H10 microneedle. Interestingly, the P25H5 microneedles exhibited faster dissolution within 45 min, while the P20H10 microneedles began dissolving after 15 min, and even after 45 min, there were a few remaining needles. The SEM micrographs of the P25H5 and P20H10 microneedles containing *Oryza sativa* L. extract complex possessed excellent morphological structure. Importantly, the mechanical test revealed that the compression force of the P25H5 and P20H10 significantly decreased when *Oryza sativa* L. extract complex was added. The P25H5-O microneedles exhibited significantly better ability to insert into skin than the P20H10-O microneedles. From these results, the P25H5-O microneedles were selected for further investigation. The percentage of drug-loading content in the microneedles was 92.95 ± 9.04%. This microneedle formulation appeared to penetrate the skin more via the transfollicular route than the solution formulation. Since the P25H5-O microneedles showed high cell viability on keratinocyte cells, this dissolving microneedle containing *Oryza sativa* L. extract complex was a promising device for transfollicular delivery through skin with safety and efficacy.

## Figures and Tables

**Table 1:** Formulations of PVP K90/HPMC E50 microneedles.

Formulations	Composition		
	PVP K90 (g)	HPMC E50 (g)	DI Water/Anhydrous Ethanol Solution (g)
P25H5	25	5	20
P20H10	20	10	20
P15H15	15	15	20
P10H20	10	20	20
P5H25	5	25	20

**Table 2:** Height, % accuracy of height, width, and % accuracy of width of PVP K90/HPMC E50 microneedles.

Formulations	Height (µm)	Accuracy of Height (%)	Width (µm)	Accuracy of Width (%)
P25H5	1479.42 ± 19.06 ^a^	98.63 ± 1.27 ^a^	456.36 ± 7.41 ^a^	101.41 ± 1.65 ^a^
P20H10	1475.01 ± 18.06 ^a^	98.33 ± 1.20 ^a^	391.18 ± 7.84 ^b^	86.93 ± 1.74 ^b^
P15H15	870.86 ± 500.00 ^b^	58.06 ± 33.33 ^b^	326.14 ± 38.25 ^c^	72.07 ± 8.50 ^c^
P10H20	393.63 ± 237.33 ^bc^	26.24 ± 15.82 ^bc^	383.98 ± 28.77 ^bc^	85.33 ± 6.39 ^bc^
P5H25	326.12 ± 32.81 ^c^	21.74 ± 2.19 ^c^	377.29 ± 148.23 ^c^	83.84 ± 32.94 ^abc^

Different superscript letters indicate significant differences (*p* < 0.05).

**Table 3:** Mechanical characteristic and penetration of the microneedles.

Formulation	Force/Array (N)	Force/Needle (N)	Height Change (%)	Blue Dot (%)
P25H5	41.94 ± 3.06 ^a^	0.19 ± 0.01 ^a^	3.40 ± 2.04% ^a^	98.52 ± 1.43 ^a^
P20H10	31.60 ± 1.60 ^b^	0.14 ± 0.01 ^b^	7.89 ± 1.74% ^b^	95.26 ± 2.45 ^b^

Different superscript letters indicate significant differences between each formulation (*p* < 0.05).

**Table 4:** Dissolution of the P25H5 and P20H10 microneedles at 0, 5, 15, 30, and 45 min using neonatal porcine skin saturated with PBS, pH 7.4.

Formulation	Time (min)				
	0	5	15	30	45
P25H5	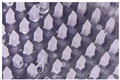	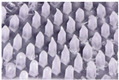	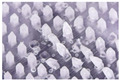	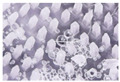	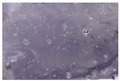
P20H10	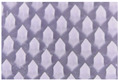	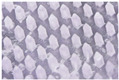	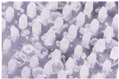	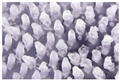	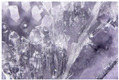

**Table 5:** Morphology and characteristics of the P25H5-O and P20H10-O microneedles.

Formulation	Height (µm)	Accuracy of Height (%)	Width (µm)	Accuracy of Width (%)	Force/Array (N)	Force/Needle (N)	Height Change (%)	Blue Dot (%)
P25H5-O	1417.26 ± 6.49 ^a^	94.48 ± 0.43 ^a^	456.63 ± 6.46 ^a^	101.47 ± 1.44 ^a^	26.52 ± 1.29 ^a^	0.12 ± 0.01 ^a^	8.22 ± 1.82 ^a^	93.04 ± 7.03 ^a^
P20H10-O	1415.29 ± 15.58 ^a^	94.35 ± 1.04 ^a^	411.45 ± 16.55 ^b^	91.43 ± 3.68 ^b^	17.75 ± 2.03 ^b^	0.08 ± 0.01 ^b^	16.30 ± 3.66 ^b^	53.63 ± 13.31 ^b^

Different superscript letters indicate significant differences between each formulation (*p* < 0.05).

**Table 6:** The cumulative amounts (ng/cm^2^), fluxes (ng/cm^2^/min), and follicular penetration per hair follicle (ng/HF) by the follicular closing technique using Franz diffusion cells at 15, 30, and 45 min of linoleic acid from the microneedle and solution sample of the extract.

Formulations	Time (min)	Cumulative Amounts (ng/cm^2^)	Fluxes (ng/cm^2^/min)	Follicular Penetration per Hair Follicle (ng/HF)
Microneedle	15	3402.87 ± 504.80 ^a^	226.86 ± 33.65 ^a^	57.36 ± 8.51 ^a^
	30	4949.63 ± 134.61 ^b^	164.99 ± 4.49 ^a,b^	98.44 ± 2.68 ^b^
	45	6924.72 ± 33.65 ^c^	153.88 ± 0.75 ^b^	144.20 ± 0.70 ^c^
Solution	15	1808.52 ± 67.31 ^a^	120.57 ± 4.49 ^a^	30.49 ± 1.13 ^a^
	30	3069.72 ± 235.57 ^b^	102.32 ± 7.85 ^b^	61.05 ± 4.68 ^b^
	45	3878.80 ± 33.25 ^c^	86.20 ± 0.55 ^b,c^	80.77 ± 0.53 ^c^

Difference in superscripts a, b, and c imply significant differences at *p* < 0.05 in 15, 30, and 45 min of the same formulations.

## Data Availability

The authors confirm that the data are contained within the article.
